# Potentially Toxic Elements Pollution in Urban and Suburban Environments II

**DOI:** 10.3390/toxics13110920

**Published:** 2025-10-27

**Authors:** Ilaria Guagliardi

**Affiliations:** National Research Council of Italy—Institute for Agriculture and Forest Systems in the Mediterranean (CNR-ISAFOM), Via Cavour 4/6, 87036 Rende, Italy; ilaria.guagliardi@cnr.it

## 1. Introduction

Following the success of the first edition of the Special Issue “Potentially Toxic Elements Pollution in Urban and Suburban Environments”, which gathered 13 high-quality contributions from international research groups, this second edition has once again proved to be a scientific success, with an equivalent number of published papers. The renewed interest and engagement of the scientific community clearly reflect the growing importance of monitoring and understanding pollution by potentially toxic elements (PTEs) as a serious and widespread environmental challenge.

Over the past few decades, accelerated population growth, rapid industrialization, urbanization, and changes in land use have dramatically increased the release and redistribution of inorganic contaminants into the environment [1,2,3,4]. The resulting contamination by PTEs, such as lead, cadmium, arsenic, chromium, and mercury, has become a global concern because of their persistence, non-biodegradability, and potential to bioaccumulate in living organisms [5,6,7]. Once introduced into the environment, PTEs can migrate across environmental matrices, air, water, soil, and biota [8,9,10,11]. They can be transferred to humans through dermal contact, inhalation of dust, and ingestion of contaminated food or drinking water [12,13]. Their presence, even at trace concentrations, may exert toxic effects on plants, animals, and humans, ultimately threatening the stability of ecosystems and public health [14,15,16].

Due to their ubiquity and complex environmental behavior, PTEs present substantial challenges for monitoring, risk assessment, and remediation [17,18]. Understanding their sources, transport mechanisms, and interactions within and between environmental compartments requires an interdisciplinary approach combining environmental geochemistry, soil and water sciences, and ecotoxicology [19]. These disciplines, supported by geostatistical and geochemical modeling, play a crucial role in identifying contamination sources, whether geological or anthropogenic, quantifying exposure risks, and informing sustainable management and policy decisions [20,21].

The present Special Issue aims to collect and compare case studies from diverse geographical contexts to advance our understanding of the behavior, transport, fate, and ecotoxicological implications of PTEs in both urban and suburban settings. The selected contributions address a broad spectrum of issues, including the assessment of soil, water, and food contamination, the evaluation of human and ecological risks, the development of analytical and geostatistical tools for source identification, and the use of bioindicators and model organisms to evaluate exposure. Together, they highlight the global dimension of PTE pollution and the need for harmonized strategies to prevent, mitigate, and monitor contamination across environmental matrices.

## 2. An Overview of Published Articles

Recent scientific contributions collected in this issue provide a comprehensive and global perspective on the pervasive challenge of environmental contamination by potentially toxic elements (PTEs), agrochemicals, and natural emissions, as well as their multifaceted implications for ecosystems, food safety, and human health. The studies span diverse geographical, environmental, and socio-economic contexts, collectively advancing our understanding of contamination sources, exposure pathways, and analytical methodologies for monitoring and risk assessment.

In Lisbon (Portugal), Arán et al. (contribution 1) examined soils from urban and rural kitchen gardens to assess fertility and contamination by trace metals. Despite elevated concentrations of Cr, Ni, and Cu exceeding regulatory thresholds, the low bioavailable fractions of these elements suggest limited ecological and health risks. The authors highlighted the importance of regular monitoring to ensure the long-term safety of urban agriculture, a growing practice driven by sustainability and food security goals.

In northern Thailand, Santha et al. (contribution 2) investigated the legacy of unregulated zinc mining in the Mae Tao watershed, resulting in widespread soil and water contamination by Cd, Zn, Pb, and Mn. Controlled leaching experiments revealed that ionic strength, rather than pH, plays a dominant role in governing metal mobility, with Cd identified as the only element posing a carcinogenic risk. This study provides valuable insights into the geochemical behavior of heavy metals in tropical agroecosystems, highlighting the need for risk-based remediation and long-term environmental monitoring.

In southwestern China, Yang et al. (contribution 3) applied hydrochemical and isotopic analyses to trace nitrate contamination in urban groundwater. Fertilizers, sewage, and livestock manure were identified as major contributors to NO_3_^−^ pollution, with nearly one-fifth of samples exceeding the national drinking water limit. Nonetheless, the overall water quality remained within acceptable ranges. The study demonstrates the utility of isotopic fingerprinting in disentangling natural and anthropogenic influences on groundwater quality in rapidly urbanizing environments.

Agricultural systems located in volcanic regions illustrate the dual role of natural processes as both stressors and stimulants of plant metabolism. In the Cotopaxi region of Ecuador, Mihai et al. (contributions 4 and 5) found that volcanic ash deposition significantly enhanced antioxidant activity and the accumulation of bioactive compounds in potatoes, corn, and beans. However, these metabolic benefits were accompanied by shifts in elemental composition and the presence of trace heavy metals, raising potential food safety concerns. The findings illustrate how volcanic inputs can simultaneously enrich and challenge agroecosystem sustainability, highlighting the importance of balancing nutraceutical gains with toxicological vigilance.

Analytical rigor remains central to contamination studies, as shown by Guagliardi et al. (contribution 6) in southern Italy, who evaluated the comparative performance of X-ray fluorescence (XRF) and inductively coupled plasma mass spectrometry (ICP-MS) for PTE determination in soils. The study identified systematic differences between methods, especially for elements such as V and Zn, emphasizing that instrument sensitivity, calibration, and matrix effects critically influence measurement reliability. These results reinforce the need for harmonized analytical protocols to ensure data comparability in environmental monitoring.

Several investigations addressed contamination pathways and biological indicators. In Zacatecas (Mexico), Ávila Vázquez et al. (contribution 7) discovered extremely high Pb concentrations in apples cultivated near mine tailings, exceeding international food safety limits by several orders of magnitude. The contamination pathway was identified as foliar absorption rather than root uptake, underscoring the vulnerability of crops in mining-impacted landscapes.

Similarly, in the Comarca Lagunera region (Mexico), Ocampo-Lopez et al. (contribution 8) found elevated Pb levels in pigeon tissues, particularly in bones, confirming their value as bioindicators of urban and industrial pollution.

A complementary methodological study by Zou et al. (contribution 9) in Changchun (China) improved the accuracy of portable XRF data through matrix correction models, providing a cost-effective and rapid approach for large-scale urban soil quality assessments.

Exposure to contaminants within indoor and aquatic environments was also explored. Somsunun et al. (contribution 10) analyzed household dust from Chiang Mai and Lamphun (Thailand) and found higher as concentrations in rural homes and elevated Cd levels in urban ones. Children were identified as the most vulnerable group, facing both carcinogenic and non-carcinogenic risks, thereby calling for stronger indoor air quality management.

In South Africa, Mugudamani et al. (contribution 11) detected herbicides, especially simazine, atrazine, and terbuthylazine, in rivers, reservoirs, and drinking water. The results revealed significant ecological risks and potential carcinogenic effects, underscoring the importance of integrated pesticide management and enhanced water treatment practices.

At a broader toxicological scale, Notario-Barandiaran et al. (contribution 12) conducted a scoping review of 77 epidemiological studies examining the neuropsychological effects of arsenic exposure during pregnancy and childhood. The majority reported adverse cognitive and psychomotor outcomes, although limitations in exposure speciation and study design hinder definitive causal inference. This synthesis highlights critical gaps in current exposure assessment and the need for longitudinal, mechanistic research.

Conversely, Al-Raddadi et al. (contribution 13) provided a reassuring perspective by analyzing the medicinal plant Boerhavia elegans, demonstrating that essential micronutrients such as Fe, Mn, and Zn were abundant. At the same time, toxic elements remained below WHO safety limits, thereby supporting its continued use in traditional medicine.

Collectively, these studies reveal the global interconnectedness of environmental contamination issues, transcending climatic, economic, and geographic boundaries. They collectively emphasize the importance of multidisciplinary approaches combining geochemical, biological, and analytical perspectives. Furthermore, they point to a shared need for improved methodological standardization, continuous environmental monitoring, and the development of science-based policies to safeguard ecosystem integrity, agricultural productivity, and public health.

## 3. Future Perspectives and Concluding Remarks

The research contributions collected in this Special Issue clearly demonstrate that contamination by potentially toxic elements remains a persistent and complex problem that transcends geographical and socio-economic boundaries. Despite considerable progress in analytical methodologies, environmental monitoring, and risk assessment frameworks, several critical gaps in knowledge still hinder the full understanding of PTE dynamics and their long-term ecological and health consequences.

Future research should focus on improving the spatial and temporal resolution of PTE monitoring through integrated, multi-scale approaches that combine field measurements, remote sensing, and geostatistical modeling. Particular attention should be paid to the interactions between PTEs and emerging contaminants, such as microplastics and nanomaterials, which may influence their mobility, bioavailability, and toxicity. Similarly, understanding the coupling between geogenic and anthropogenic sources in rapidly urbanizing regions will be essential to designing effective prevention and remediation strategies.

Advances in analytical chemistry, isotopic tracing, and in situ sensing technologies offer new opportunities to refine source apportionment and exposure assessment. These should be complemented by experimental and modeling studies addressing biogeochemical transformations and long-term sequestration processes in soils and sediments. In parallel, the integration of human biomonitoring data with environmental measurements will enhance the ability to assess real exposure pathways and cumulative risks, particularly in vulnerable populations such as children and communities living near industrial or mining areas.

From a policy perspective, there is a pressing need to harmonize environmental standards and monitoring protocols across countries, ensuring data comparability and supporting international initiatives for pollution control and health protection. Collaborative networks and open-access data platforms can play a key role in achieving this goal, promoting transparency, reproducibility, and the global sharing of best practices.

Overall, this second edition of the Special Issue “Potentially Toxic Elements Pollution in Urban and Suburban Environments” reinforces the critical importance of continued interdisciplinary research and coordinated policy actions. By combining geochemical insight with technological innovation and public health awareness, the scientific community can contribute to a more accurate understanding of PTE behavior and to the sustainable management of contaminated environments, ultimately safeguarding ecosystem integrity and human well-being.

## Data Availability

No new data were created or analyzed in this study. Data sharing is not applicable to this article.
